# Influence of Surface Properties of Nanostructured Ceria-Based Catalysts on Their Stability Performance

**DOI:** 10.3390/nano12030392

**Published:** 2022-01-25

**Authors:** Boyu Li, Eric Croiset, John Z. Wen

**Affiliations:** 1 Department of Chemical Engineering, University of Waterloo, Waterloo, ON N2L3G1, Canada; b264li@uwaterloo.ca (B.L.); ecroiset@uwaterloo.ca (E.C.); 2 Department of Mechanical & Mechatronics Engineering, University of Waterloo, Waterloo, ON N2L3G1, Canada

**Keywords:** ceria-based catalysts, surface properties, stability, cycles, nanoparticles

## Abstract

As the poor cycling stability of CeO_2_ catalysts has become the major obstacle for applications of diesel particulate filters (DPF), it is necessary to investigate how to reduce their structural and compositional changes during soot oxidation. In this study, different ratios of Samarium (Sm) were doped into the lattice of CeO_2_ nanoparticles to improve the catalytic performance as well as surface properties. The stability was investigated by recycling the catalyst, mixing it with soot again, and repeating the thermogravimetric analysis (TGA) tests seven times. Consistent observations were expected for more cycles. It was found that doping 5%, 10%, and 20% samarium into the CeO_2_ lattice can improve the catalyst stability but at the cost of losing some activity. While the catalyst became more stable with the increasing Sm doping, the 10% Sm-doped catalyst showed the best compromise between stability and activity. Ce^3+^ and O_α_ were found to play important roles in controlling catalytic soot oxidation activity. These two species were directly related to oxygen vacancies and oxygen storage capacity of the catalyst. Sm-doped catalysts showed a minimized decrease in the Ce^3+^ and O_α_ content when the fresh and spent catalysts were compared.

## 1. Introduction

Ceria has already been widely studied in soot oxidation because of its excellent redox properties and oxygen storage capacity (OSC). Previous studies have shown that metal-doped ceria can further improve the activity of ceria-based catalysts [1]. In a previous study that investigated the activities of iron-doped ceria catalysts at different doping ratios, we showed 5% iron doping prepared with the solution combustion synthesis (SCS) method performed best due to its highly reactive Fe–O–Ce sites [2].

It was reported that the active sites, crystal structures, and surface properties of ceria-based catalysts could change during the reactions, resulting in poor stability of ceria-based catalysts [3]. All studies acknowledged that pure CeO_2_ shows poor stability for soot oxidation, which has become a major obstacle for the wide-ranging applications of ceria-based catalysts [3,4,5,6]. Liang et al. [3] have studied the thermal stability of CeO_2_ for soot oxidation by aging the catalyst at 800 °C for 20 h in air. They found that the temperature corresponding to the maximum rate for CO_2_ production with an aged catalyst increased by about 60 °C compared to a fresh catalyst because of the highly reduced surface area and sintering. Aneggi et al. [4] observed that an aging CeO_2_ catalyst at 750 °C for 12 h in air decreased its activity for soot oxidation as it lost oxygen storage capacity. Liu et al. [5] investigated the deactivation of CeO_2_ for soot oxidation through isothermal conditions at 300 and 350 °C; they found that deactivation occurred at isothermal conditions and became more severe at higher temperature. The main reported reason for deactivation is inefficient $O_{2}^{-}$ formation. Corro et al. [6] tested the stability of CeO_2_ towards soot oxidation during six cycles and found a slow and continuous deactivation of CeO_2_.

Therefore, the stability of CeO_2_-based catalysts for soot oxidation needs to be improved. Many studies have looked at the incorporation of metal dopants into the CeO_2_ lattice to improve stability, as described next. Wu et al. [7,8,9,10] added BaO or Al_2_O_3_ into transition-metal- (Mn, Cu) doped CeO_2_ to improve its thermal stability. They found that these metals could improve the thermal stability after aging at 800 °C for 24 h in air because they hinder crystal growth. Aneggi [4] and Liang et al. [3] incorporated ZrO_2_ into Fe–Ce and Cu–Ce mixed oxides and found enhanced thermal stability due to the formation of stable solid solutions. Gao et al. [11] aged a Nd–Ag/CeO_2_ catalyst at 700 °C for 48 h under 1% O_2_/10% H_2_O/N_2_, revealing Nd could prevent crystal growth, thus improving thermal stability of Ag/CeO_2_. Xiong et al. [12] added Y and La into a Zr/CeO_2_ catalyst and aged it from 700 to 1000 °C. The catalyst activity remained stable below 800 °C but decreased significantly above 900 °C. Zhang et al. [13,14] investigated the influence of thermal stability by adding Y into MnO_x_–CeO_2_ and introducing Al, La, Y, or Zr into Pt/MnO_x_–CeO_2_ after aging at 800°C for 12 h in air. They observed that the incorporation of dopants can prevent the sintering and slow down crystal growth. Peralta et al. [15,16] introduced Ba into alkali metals-modified ceria, such as K/CeO_2,_ and obtained good thermal stability, as no deactivation was observed after aging at 800 °C. Noble metals, such as Ru, have also been investigated as dopants to successfully improve ceria-based catalyst stability after aging at 800 °C in O_2_ [17]. Although all the studies described previously investigated stability through accelerated thermal aging, only a few studies investigated stability through more realistic, albeit more cumbersome, soot oxidation cycles [18,19,20,21]. La loaded on CeO_2_–ZnO (five cycles) [21], K added into CeO_2_ (three cycles) [18], codoping of Ag and Mn into CeO_2_ (three cycles) [19], and Au-doped Ce_0.8_Zr_0.2_O_2_ (three cycles) [20] all showed enhanced stability of ceria-based catalysts for soot oxidation. These studies identified that resistance of sintering and crystal growth, ability of CO_2_ desorption, and oxygen-species replenishment play important roles regarding catalyst stability in soot oxidation.

The stability of ceria-based catalysts has also been investigated for applications other than soot oxidation [22,23]. One family of applications relates to catalytic combustion of different gases. Dai et al. [24] investigated the stability and deactivation of the CeO_2_ catalyst and found that T50 increased from 165 °C to 325 °C after nine cycles of trichloroethylene combustion. Zhang et al. [25] studied Al-, Zr-, La-, or Y-doped Pt/MnO_x_–CeO_2_ stability toward NO oxidation after aging and observed that the modified ceria catalyst showed better activity. Han et al. [23] investigated the stability of the ZrO_2_-doped CeO_2_-based catalyst for toluene combustion, revealing that ZrO_2_ can stabilize the surface active structure, thus improving stability. Polychronopoulou et al. [26] investigated Sm_2_O_3_/CeO_2_ catalysts for CO oxidation and found that adding Sm can significantly improve the thermal stability of the conventional CeO_2_ catalyst. Mandal et al. [27] have demonstrated enhanced stability of Gd–Sm–CeO_2_ for benzyl alcohol oxidation. Another important family of applications using ceria-based catalysts is in solid-oxide fuel cells (SOFCs) (and the reverse operation, solid-oxide electrolysis, SOEC) for intermediate temperatures (650–750 °C). The main ceria-based material investigated for SOFC and SOEC are samarium-doped ceria (SDC) [28,29] and gadolinium-doped ceria (GDC) [22,30]. Sm and Gd were essentially added to stabilize ceria during the operation at high temperature, while achieving good oxygen-ion conductivity, as they can inhibit crystal growth and prevent sintering [31].

From the above literature review on the stability of the ceria-based catalyst, it is somewhat surprising to see almost no studies on samarium-doped ceria despite proven long-term stability at high temperatures (750–850°C) in SOFC/SOEC. Granted, SDC in SOFC/SOEC has a different purpose (electrolyte to transport oxygen ion) than it would have in soot oxidation. Yet, SDC should be a promising stable catalyst for soot oxidation because of its thermal stability at high temperature. Very few papers studied Sm as a dopant to ceria for soot oxidation. Liu et al. [32], using a loose-contact condition, studied activity and thermal stability (calcination at 800 °C for 20 h) of a 20% Sm-doped ceria catalyst prepared through microwave-assisted heating decomposition. Sudarsanam et al. [33] carried out a similar study, except under a tight-contact condition and with the coprecipitation catalyst preparation method. Both reported increases in combustion temperatures after aging but are not conclusive regarding actual catalyst stability. Anantharaman et al. [34] studied the effect of Sm content on soot-oxidation reactivity for the Sm-doped ceria catalyst prepared by the EDTA-citrate method but did not investigate catalyst stability; they found that 10% Sm doping performed best on a fresh catalyst calcined at 600 °C for 5 h.

The present study aims to develop an optimum ratio of Sm doping into the CeO_2_ catalyst to improve catalyst stability for soot oxidation. Therefore, different ratios of Sm doping were investigated along with its influence on catalyst surface properties and activity. Characterizations were performed to understand surface and crystal properties of catalyst. Seven repeating reaction cycles were used to study the activity and stability of the Sm-doped CeO_2_ catalyst.

## 2. Materials and Methods

### 2.1. Catalysts’ Preparation

Sm_x_/Ce_1−x_ (x = 0.05, 0.10, and 0.20 in percent molar ratio) catalysts were prepared by the solution combustion synthesis (SCS) method. An aqueous solution of Samarium nitrate nonahydrate (Sigma-Aldrich, St. Louis, MO, USA, CAS: 13759-83-6, 99.999% trace metals basis), cerium nitrate hexahydrate (Sigma-Aldrich, CAS: 10294-41-4, 99.999% trace metals basis), and glycine (Sigma-Aldrich, CAS: 56-40-6) in a stoichiometric ratio was mixed under vigorous stirring at 90 °C to form a gel. The gel was then combusted on a heating plate. The combustion procedure was fast, producing nano powders (around 20 nm). The resulting samples were then calcined at 500 °C for 5 h. The pure CeO_2_-SCS catalyst was developed using a similar procedure.

### 2.2. Catalysts’ Activity and Stability Tests

Thermogravimetric analysis (TGA) was used to test the catalyst’s activity for soot oxidation through a TA Instrument Q500 apparatus (TA Instruments, New Castle, DE, USA). Printex-U carbon black was used as the model of soot. Catalyst and soot particles were weighted at a ratio of 9:1 to make sure soot can be fully oxidized, after a number of trials. They were then mixed by grinding them for 10 min to obtain a tight-contact condition [35]. For the first cycle of the TGA test, a weighted amount of a 40 mg sample was pretreated at 150 °C under 60 mL/min nitrogen for 30 min to remove water. Then, the sample was heated up to 600 °C under 40 mL/min air with a heating rate of 10 °C/min. The thermogravimetric curves were obtained by continuously recording the mass change, along with increased temperature. Soot oxidation in the absence of catalysts was performed for the comparison purpose. Note that from our past observations, heating the carbon-catalyst mixture to 600 °C is sufficient to achieve the soot conversion rate of 100% for ceria catalysts, while the pure carbon sample is completely oxidized after the temperature reaches 700 °C [2]. The catalyst’s activity was evaluated by T50. T50 is the combustion temperature, identified as the temperature when 50% of soot is oxidized [36,37,38,39].

The stability tests were performed through several soot oxidation cycles using the same catalyst. One cycle is defined by a step of mixing soot and catalyst particles, followed by a step of soot oxidation in the TGA. Once the first cycle soot-oxidation reaction was completed, the remaining catalyst was collected and remixed with soot particles again under a tight-contact condition with the same weighting ratio of 9:1. The TGA test was run again with the newly mixed sample. This procedure was repeated for several cycles. Note that after each cycle, it was inevitable that some amount of the catalyst is lost (mostly during the mixing step where several particles stick to the mortar and pestle). This means that for a given amount of fresh catalyst there will be a maximum number of cycles that can be investigated; for example, with our typical 36 mg of fresh catalyst (with 4 mg of carbon), it was hardly possible to go beyond seven cycles.

### 2.3. Characterization of the Catalysts

An X-ray powder diffraction (XRD) analysis was carried out using an X-ray powder diffractometer (German Bruker D4 (40 kV, 30 mA), Bruker, Billerica, MI, USA with a position-sensitive detector and CuKα radiation). The XRD patterns were recorded from 5° to 85° in steps of 0.01°. The XRD patterns were analyzed based on the Powder Data File database (International Centre of Diffraction Data, Newtown Square, PA, USA, accessed on: 1 September 2021). Specific surface areas were evaluated by N_2_ adsorption–desorption isotherms on a Beishide 3H-2000PS2 static-volumetric method analyzer, BEISHIDE Instrument Technology, Beijing, China. The Brunauer-Emmett-Teller (BET) method was used to calculate the catalysts’ surface areas. The catalysts’ morphologies and microstructures were characterized by a Field-emission scanning electron microscope (FE-SEM, Zeiss MERLIN with Gemini-II column, Zeiss, Oberkochen, Germany). The X-ray photoelectron spectroscopy (XPS) analysis was conducted on the ESCALab220i-XL electron spectrometer (VG Scientific Ltd., East Grinstead, UK) with a 300 W AlKα X-ray source to study the oxidation states and oxygen species on the catalysts’ surfaces. Binding energy was calibrated by standard C1s peaks at 284.8 eV. XPS peaks was analyzed using CasaXPS software, Version 2.3.24, Clearwater, FL, USA. The Raman spectra of catalysts were measured on a Renishaw inVia micro laser Raman spectrometer (Renishaw plc, Wotton-under-Edge, UK) with a 4 mW Ar^+^ laser source (λ_ex_ = 532 nm) and a cooled CCD detector at room temperature to distinguish chemical structures. The scanning range was 200–800cm^−1^ with a 60 s acquisition time.

## 3. Results and Discussion

### 3.1. Fresh and Spent Catalysts’ Characterizations

**Figure 1:**
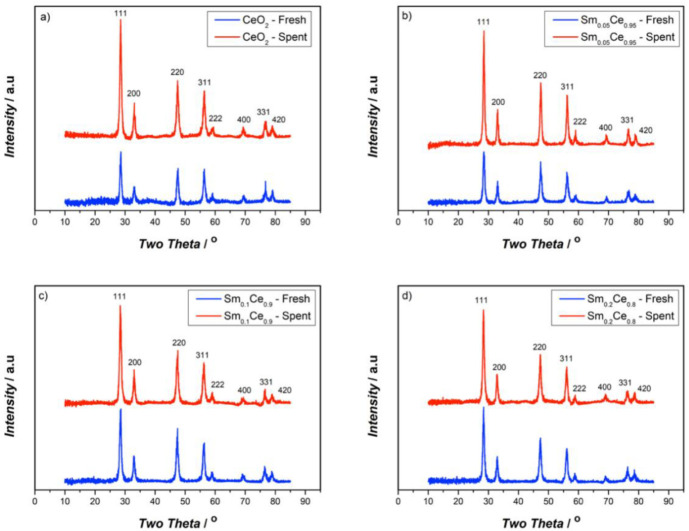
XRD results of fresh and spent catalysts after five cycles of reactions: (**a**) CeO_2_, (**b**) Sm_0.05_Ce_0.95_, (**c**) Sm_0.1_Ce_0.9_, (**d**) Sm_0.2_Ce_0.8_.

The crystalline structures of catalysts were studied by XRD. Figure 1 presents the XRD pattern of fresh and spent Sm-doped CeO_2_ catalysts, as well as pure CeO_2_ for comparison. All XRD patterns show similar main diffractions peaks, which can be attributed to (111), (200), (220), (311), (222), (400), (331), and (420) planes. These peaks refer to typical cubic fluorite structures of pure CeO_2_ [40]. No peaks referring to Sm_2_O_3_ were found, even for 20% SDC, meaning that no individual Sm_2_O_3_-crystal structure was detected, indicating that the Sm forms a solid solution in the ceria lattice. For fresh catalysts, when increasing the Sm-doping ratio, the characteristic peaks shift to the lower 2θ diffraction angles, further suggesting that Sm is incorporated into the CeO_2_ lattice and forms a solid solution. The unit-lattice parameters were calculated by Bragg’s law using the strongest peak (111), and the crystallite size was calculated by the Scherrer equation. With an increase in Sm-doping, the lattice parameter (as shown in Table 1) tends to increase because of the larger Sm (1.07 Å) substituting smaller ceria (0.97 Å) in the lattice, thus resulting in lattice expansion [41].

As seen in Table 1, the comparison of fresh and spent catalysts shows that the lattice parameters of spent catalysts slightly increase compared to those of fresh catalysts with the same doping ratio. Although those increases are very small, the trend is clear that the change in lattice parameters decreases as the amount of Sm increases: for pure ceria, 5%, 10%, and 20% Sm; the differences in lattice parameters between fresh and spent catalysts are 0.013, 0.006, 0.004 and 0.003 Å, respectively. While for fresh catalysts, the crystallite size clearly increases as the amount of Sm increases (from 12.3 nm for pure ceria to 13.6 nm for Sm_0.2_Ce_0.8_); such a clear trend was not observed for the spent catalysts where the crystallite size seems to converge for the size range of between 13.5 and 14.5 nm. The spent catalysts always show higher crystallite sizes than the fresh ones, indicating that there is an increase in crystallinity after several cycles of reactions on the catalyst. For all spent catalysts, Sm_0.1_Ce_0.9_ notably shows the lowest crystallite size.

The BET surface areas for Sm-doped ceria catalysts are listed in Table 1. For the fresh Sm-doped catalysts, except Sm_0.1_Ce_0.9_, they all have a surface area of 41–42 m^2^g^−1^, which are lower than that of fresh pure CeO_2_ (45.5 m^2^g^−1^). The fresh Sm_0.1_Ce_0.9_ catalyst shows the highest BET surface area of 48 m^2^g^−1^. When comparing fresh and spent catalysts, the BET surface area of pure CeO_2_ decreases the most (from 45.5 to 40.9 m^2^g^−1^). Sm-doped CeO_2_ catalysts also show a slight decline in the BET surface area (2–4% loss), regardless of the doping ratio. Interestingly, except for Sm_0.1_Ce_0.9_, all other spent catalysts, including pure ceria, have similar surface areas at around 40–41 m^2^g^−1^. There seems to be something particular for Sm_0.1_Ce_0.9_, which has the highest surface area for both fresh and spent catalysts. Note that this catalyst also stands out, having the lowest crystallite size after several cycles. Since the catalysts for XRD and BET characterization were made from different batches, it indicates that Sm_0.1_Ce_0.9_ presents interesting properties, which cannot be attributed to experimental error.

**Figure 2:**
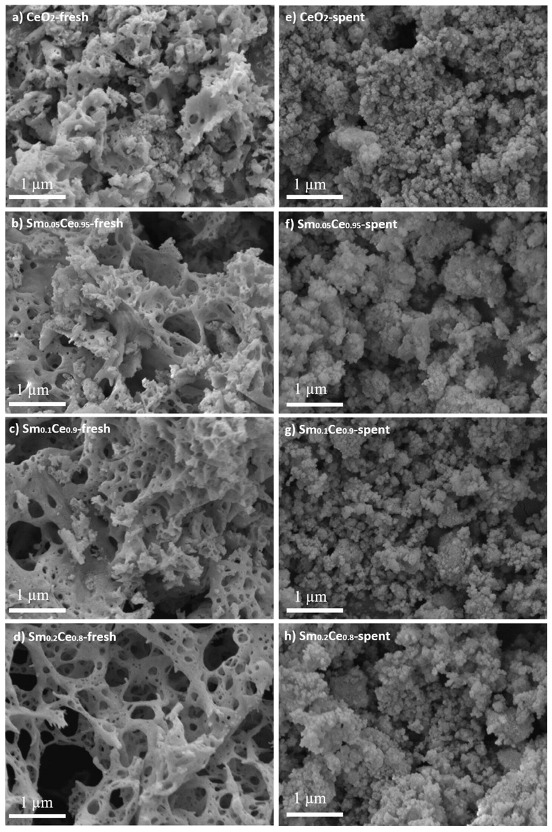
SEM images of all fresh and spent catalysts after five cycles: (**a**) CeO_2_ fresh; (**b**) Sm_0.05_Ce_0.95_ fresh; (**c**) Sm_0.1_Ce_0.9_ fresh; (**d**) Sm_0.2_Ce_0.8_ fresh; (**e**) CeO_2_ spent; (**f**) Sm_0.05_Ce_0.95_ spent; (**g**) Sm_0.1_Ce_0.9_ spent; (**h**) Sm_0.2_Ce_0.8_ spent.

An SEM was used to study the morphologies of the produced catalysts. Figure 2 shows the SEM images of all fresh and spent catalysts. All fresh catalysts have spongy structures with large openings, which are due to their fast combustion reaction during the SCS procedure. The main effect of Sm doping on the catalyst morphology is to increase those openings. However, all spent catalysts lose the spongy structure and tend to agglomerate and in the end have very similar morphologies.

**Figure 3:**
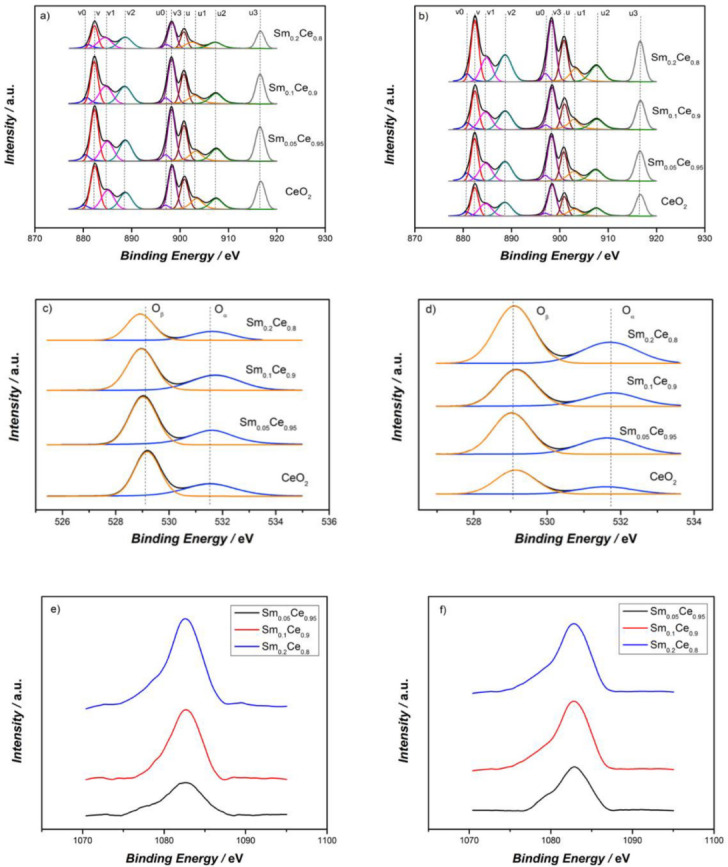
XPS results of (**a**) Ce3d of fresh catalysts, (**b**) Ce3d of spent catalysts, (**c**) O1s of fresh catalysts, (**d**) O1s of spent catalysts, (**e**) Sm 3d5/2 of fresh catalysts, (**f**) Sm 3d5/2 of spent catalysts.

XPS was used to detect different oxidation states of each element. Figure 3 is the XPS spectra of all catalysts regarding their Ce3d, O1s, and Sm 3d5/2 spectra. The binding energies of XPS spectra were calibrated using the C1s peak at 284.8 eV. C1s spectra of all catalysts are provided in the Supplementary Materials. While performing the Ce3d deconvolution, the FWHM (full width at half maximum) was set to a narrow range of 2–2.7 eV, and the peak area of 3d5/2 to 3d3/2 was set to 3:2. Ce3d spectra can be split into 10 peaks for detailed analysis. The peaks labeled with “u” and “v” correspond to the spin-orbit splitting of Ce 3d5/2 and Ce 3d3/2, respectively. These peaks, v, v2, v3, u, u2, and u3, are the characteristic peaks for the Ce^4+^ species, and the peaks noted with v0, v1, u0, and u1 correspond to the Ce^3+^ species [42,43,44,45,46]. Detailed peak positions are provided in the Supplementary Materials. It is clear that ceria exists primarily as Ce^4+^ for each catalyst, with the coexistence of some Ce^3+^. Since oxygen vacancy is generated when Ce^4+^ is reduced to Ce^3+^, it is crucial to calculate the Ce^3+^ percentage to evaluate the generation of oxygen vacancy on the catalyst surface [37,46]. The calculations of the integrated peak areas can be used to quantitatively analyze XPS results. The ratio of Ce^3+^ was calculated by dividing the peak areas of Ce^3+^ by the total peak area. Table 2 shows the results of the Ce^3+^ percentage for fresh and spent catalysts. For fresh catalysts, it can be found that adding Sm into the CeO_2_ lattice decreases the Ce^3+^ percentage (about 17–19%) from that in pure ceria (24.2%). The 5% and 10% Sm doping possess similar Ce^3+^ percentages (18.8% and 19.3%) and the 20% Sm-doped catalyst has the lowest Ce^3+^ percentage (17.3%). Table 2 also shows that the Ce^3+^ percentage of the spent catalysts decreases when compared to fresh catalysts. Pure CeO_2_ in particular, shows a significant Ce^3+^ percentage decrease from 24.2% to 15.8%. In other words, it changes from the highest percentage to the lowest one. However, Sm-doped catalysts show a lower decrease (0.9% for 5% doping and 0.8% for 10% doping). The Ce^3+^ percentage for Sm_0.2_Ce_0.8_ catalysts only decreases by about 0.5%. Those results suggest that Sm helps establish a more stable catalyst surface, at least in terms of the Ce^3+^ content.

The O1s spectra were curve fitted into two peaks, including lattice oxygen, O_β_, at a lower binding energy of 529.5 eV and the surface oxygen species, O_α_, at a higher binding energy of 531.7 eV [47]. The concentration of O_α_ is critical to evaluate the oxygen storage capacity (OSC) and the potential active oxygen for soot oxidation. The ratio, O_α_/(O_α_+ O_β_), was calculated by dividing the peak areas of O_α_ by the total peak areas, whose results are shown in Table 2. This table shows that for fresh catalysts, Sm doping up to 10% marginally decreases the O_α_ ratio (from 39.6% down to 39.1%). However, the decrease in the O_α_ ratio is more pronounced for 20% Sm doping (O_α_ ratio of 34.2%). The results regarding the O_α_ ratio are in agreement with that of the Ce^3+^ percentage. Regarding spent catalysts, the O_α_ ratio of pure CeO_2_ drops significantly compared to that of the fresh CeO_2_ catalyst (from 39.6% to 29.5%) and becomes the lowest among all spent catalysts. On the other hand, the O_α_ percentage for 5% and 10% Sm-doped ceria catalysts decreases by less than 2 percentage points. Finally, the 20% Sm-doped catalysts do not show a decrease in O_α_ content, although its final value is still lower than that of the other two Sm-doped ceria catalysts. Those results suggest that Sm doping considerably reduces the loss in surface oxygen after several cycles.

The peaks around 1082.6 eV in the Sm 3d5/2 spectra confirm that Sm is present as a +3-oxidation state in the catalyst, which agrees with the literature [48,49]. The surface ratio of Sm/Ce was calculated through XPS results. For fresh catalysts, the ratios are 0.059, 0.122, and 0.342, for 5%, 10%, and 20% Sm doping, respectively. Compared to the stoichiometric ratios of 0.053, 0.111, and 0.25 for 5%, 10%, and 20% Sm doping, respectively, the fresh catalysts show slightly higher values, indicating there is an enrichment of Sm on the detected surface of these catalysts. For spent catalysts, the Sm/Ce are 0.068, 0.138, and 0.355 for 5%, 10%, and 20% Sm doping, respectively. These ratios are greater in comparison with the fresh catalyst values, which is possibly due to Sm in the bulk catalysts tending to move to the surface of catalysts during the reactions. The 20% Sm doping shows the smallest change of Sm/Ce, suggesting a more stable catalyst surface.

**Figure 4:**
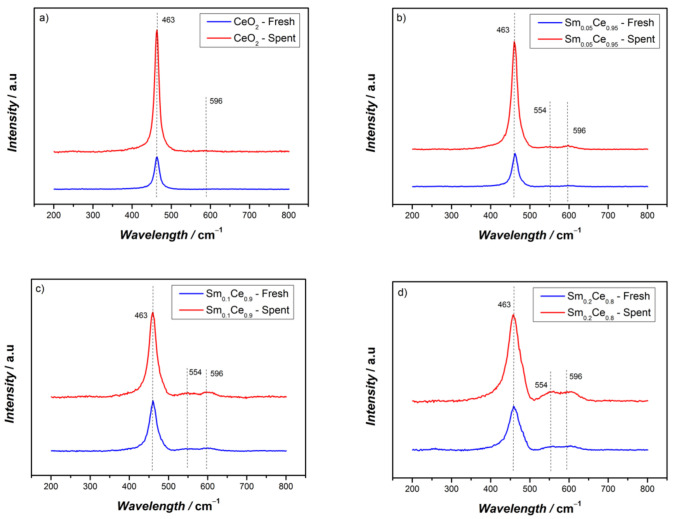
Raman results of fresh and spent catalysts after five cycles of reactions: (**a**) CeO_2_, (**b**) Sm_0.05_Ce_0.95_, (**c**) Sm_0.1_Ce_0.9_, (**d**) Sm_0.2_Ce_0.8_.

The Raman spectra of Sm-doped CeO_2_ catalysts are depicted in Figure 4. The dominant band around 463 cm^−1^ corresponds to the F2g symmetric oxygen mode within a CeO_2_ cubic fluorite structure, which is in agreement with the XRD results [33]. With an increase of the Sm doping ratio, the F2g band shifts to lower frequencies due to the cell expansion in the catalysts. No peaks of Sm_2_O_3_ (~375 cm^−1^) were found, which reinforces that Sm-Ce forms a solid solution [27]. The weak peak around 596 cm^−1^ represents the surface defects, including the intrinsic oxygen vacancies caused by Ce^3+^ in the lattice [35]. The peak around 554 cm^−1^ refers to the extrinsic oxygen vacancy created by Sm^3+^ substituting Ce^4+^ to maintain charge neutrality [50]. With an increase in Sm doping, the peak intensity at 554 cm^−1^ becomes stronger, indicating that more Sm^3+^-associated oxygen vacancies are created. The ratio, I_554_/I_596_, represents, therefore, the ratio of oxygen vacancies originating from Sm^3+^ over that from Ce^3+^. The results pertaining to the I_554_/I_596_ ratio are shown in Table 3. For fresh catalysts, as expected, the incorporation of Sm increases the I_554_/I_596_ ratio. Note that for spent Sm_0.2_Ce_0.8_, this ratio is above 1, indicating more oxygen vacancies from Sm^3+^ than from Ce^3+^, whereas it is the opposite for Sm content below 10% (values of the I_554_/I_596_ ratios below 1).

After five cycles of reactions, the I_554_/I_596_ ratio for all catalysts further increases (by about 0.1). This indicates that after several cycles, the relative amount of oxygen vacancy correlated to Sm^3+^ increases (and its corollary, the relative amount of oxygen vacancy correlated to Ce^3+^ decreases). The peak intensity of the Raman spectra increases in all cases after five cycles, possibly due to the increase in crystal growth, which is in agreement with the XRD results. Another reason could be the lattice distortion of the spent catalysts leading to resonance with the inlet Ar^+^ laser source.

### 3.2. Activity Test

**Figure 5:**
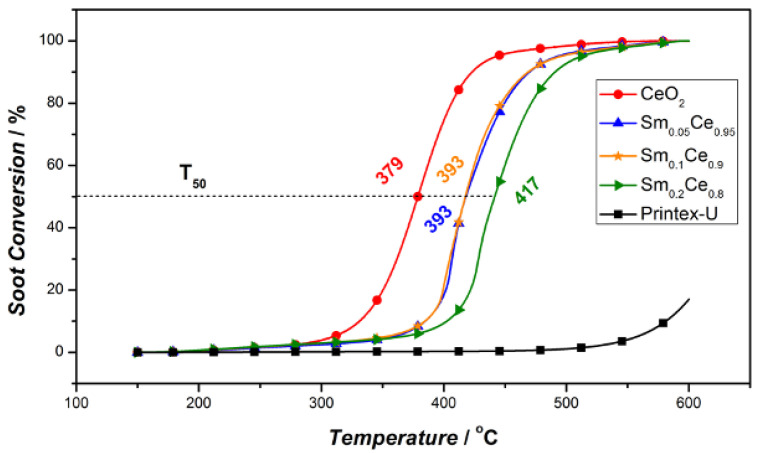
Soot conversion activity test of fresh Sm-doped ceria catalysts.

TGA experiments were performed to study the activity of the fresh Sm-doped CeO_2_ catalyst for soot oxidation. Figure 5 depicts soot conversion as a function of temperature. The fresh pure CeO_2_ catalyst achieves the highest activity, as indicated by the lowest T50. Increasing the Sm content in the fresh catalysts tends to increase T50, revealing a loss in activity. In fact, the 5% and 10% Sm-doping ratios for the fresh catalyst show similar effects on catalyst activity, whereas the 20% Sm-doping sample leads to the worst activity. In comparison, soot oxidation without the catalyst was conducted under the same reaction condition (Printex-U in Figure 5), and it shows very little soot oxidation below 500 °C, suggesting that the catalyst investigated here significantly increases soot oxidation.

Since catalytic soot oxidation is a surface sensitive reaction, i.e., reaction only happens at the contact point of soot and catalyst, the crystallite size, specific surface area, and morphology can all play important roles on the catalyst activity [1,51]. From Figure 2, the morphology does not change significantly between pure ceria and Sm-doped catalysts, and thus cannot explain the difference in reactivity. Table 1 indicates that the BET surface area increases slightly when increasing the Sm content, but this would lead to higher activity at the higher Sm content, which is the opposite of what was observed; the BET surface area, thus, cannot explain the trend in activity here. Finally, Table 1 shows a modest change in the crystallite size when increasing the Sm content and without a clear trend. Crystallite size can, therefore, also be dismissed as an explanation for the activity trend for the fresh catalysts.

Soot oxidation on ceria-based catalysts occurs via the so-called Mars–van Krevelen mechanism [1], where the active surface oxygen reacts with soot through the catalyst-soot contact point. Once the active oxygen is consumed, an oxygen vacancy is generated at the surface, and bulk O_2_ fills this vacancy and create another active oxygen. Therefore, factors affecting the amount of active surface oxygen and oxygen vacancies can also play important roles in determining catalyst activity. The Ce^3+^ content is a good representation of oxygen-vacancy generation, which, in turn, can be a potential site for creating active surface oxygen. Here, surface oxygen and oxygen vacancies are related to the Ce^3+^ content, O_α_ percentage, and I_554_/I_596_ ratio. Those are discussed next.

**Figure 6:**
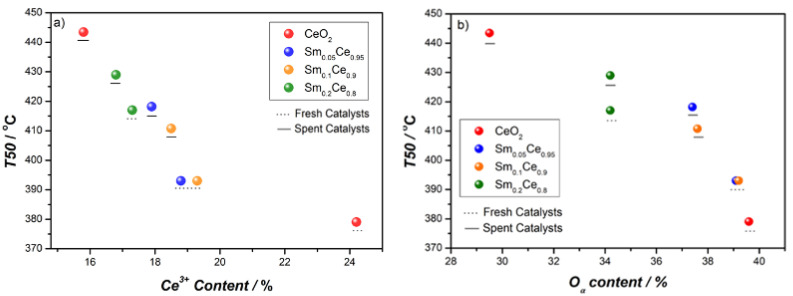
Effect of Ce^3+^ percentage and O_α_ percentage on activity for all catalysts: (**a**) T50 vs. Ce^3+^ percentage, (**b**) T50 vs. O_α_ percentage.

Figure 6a shows the relation between T50 and the Ce^3+^ percentage, which indicates that the higher the Ce^3+^ content, the lower T50 (and thus, higher the activity). The highest Ce^3+^ ratio (24.2%) corresponds to the fresh pure CeO_2_ catalyst, which can be a reason for the highest activity of pure ceria. The activity results reported in Figure 5 for Sm-doped catalysts correlates very well with the Ce^3+^ content shown in Table 2: 5%- and 10%-doped CeO_2_ have identical Ce^3+^ content and also very similar activity (e.g., same T50), whereas Sm_0.2_Ce_0.8_ has a much lower Ce^3+^ content, as well as much lower activity (i.e., higher T50). Figure 6b shows the relation between T50 and the O_α_ percentage, which follows the trend of the Ce^3+^ content, indicating that surface-oxygen species are also vital for catalyst activity. It has been reported that the higher O_α_ concentration could result in superior catalyst activity, as it can evaluate the oxygen-storage capacity and potential active oxygen for the reaction. From Table 2, the O_α_ percentage slightly decreases for 5% and 10% Sm-doped catalysts, but it considerably decreases for 20% Sm doping when comparing with pure CeO_2_; the O_α_ percentage data also correlates very well with the activity results.

**Figure 7:**
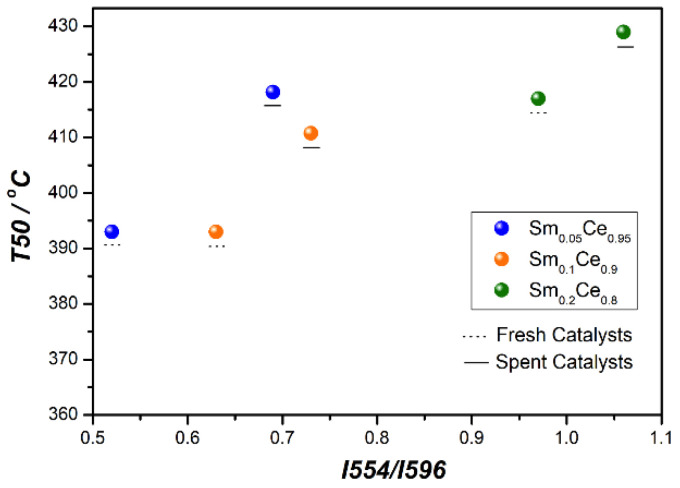
The relation between T50 and I_554_/I_596_.

The relation between T50 and the I_554_/I_596_ ratio is illustrated in Figure 7, which shows that T50 increases when the I_554_/I_596_ ratio increases. This implies that Sm^3+^-associated oxygen vacancy decreases the catalyst activity. From the XPS results, it is already known that higher Ce^3+^-associated oxygen vacancy plays a key role in improving catalyst activity. Therefore, both the increase in oxygen vacancy around Sm^3+^ and the decrease of oxygen vacancy adjacent to Ce^3+^ lead to activity decline.

### 3.3. Stability Tests for Sm-Doped CeO_2_ Catalysts

**Figure 8:**
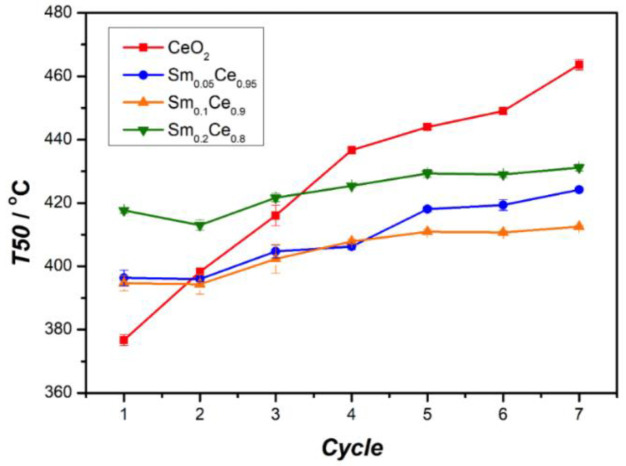
Stability of Sm-doped CeO_2_ for soot oxidation.

Figure 8 presents the results of stability on Sm-doped CeO_2_ for soot oxidation through seven cycles. This figure shows that T50 for the pure CeO_2_ catalyst increases after each cycle, going from the lowest value (379 °C) with the fresh catalyst to the highest one (462 °C) after seven cycles. The 5% Sm-doped catalyst also shows a continuous increase in T50 after each cycle but at a much lower rate than pure CeO_2_. The samples with 10% and 20% Sm doping follow very similar trends (as seen in Figure 8, they are almost parallel): first, a moderate increase in T50 after three or four cycles, followed by a near plateauing of T50. The differences in T50 between the initial and plateau values are 14 °C for 10% Sm and 8 °C for 20% Sm, respectively. Those results indicate good stability for Sm doping above 10%. As a plateau is reached after the fifth cycle, it is expected that the T50 of catalysts containing more than 10% Sm doping would have a very limited change for more cycles. Considering activity and stability together, the 10% Sm-doped catalyst performs best among all catalysts, with a stability comparable to the 20% Sm but with the lowest T50 after seven cycles (412 °C).

**Figure 9:**
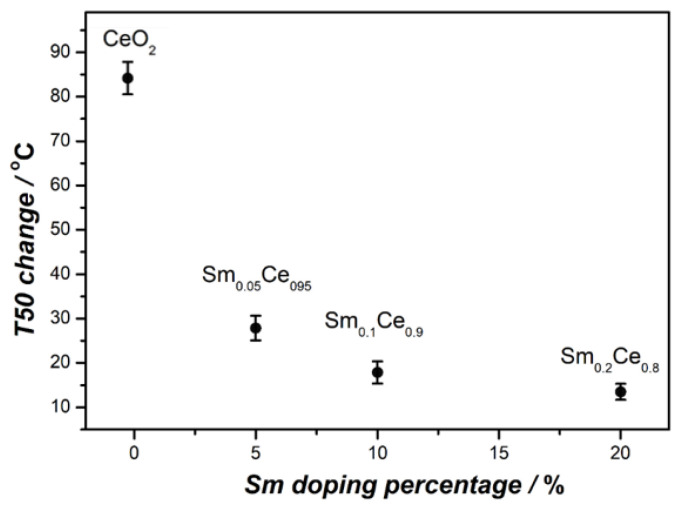
Relationship between T50 change (between first and seventh cycles) and Sm-doping ratio.

Figure 9 shows the relationship between the change of T50 (between first and seventh cycle) and the Sm-doping ratio. The spent CeO_2_ catalyst leads to an 83 °C change in T50, compared to the fresh one. T50 of 5% and 10% Sm-doped catalysts show smaller differences, which are about 27 °C and 18 °C, respectively. T50 for the 20% Sm-doped catalyst shows the lowest difference (13 °C). It is clear that the T50 difference decreases with higher Sm doping, indicating that richer Sm contributes to a more stable catalyst, albeit with lower activity.

**Figure 10:**
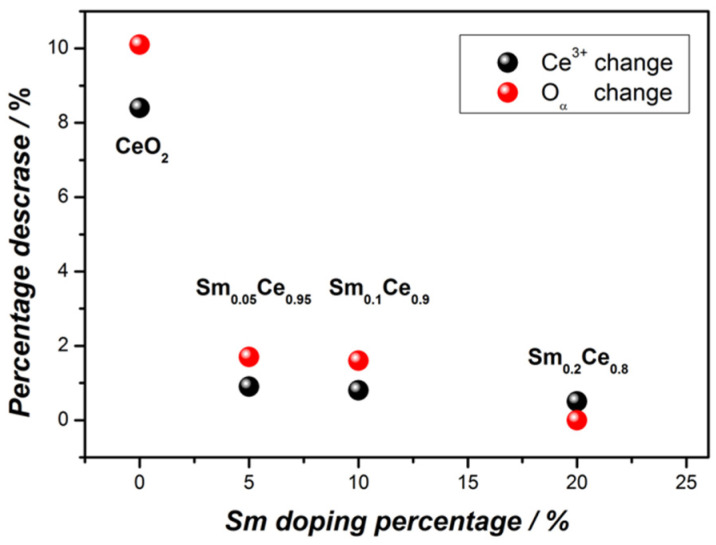
Relationship between Ce^3+^/O_α_ change and Sm-doping ratio.

As discussed previously, the Ce^3+^ content plays an important role for catalyst activity towards soot oxidation. Figure 10 illustrates the relationship between the Ce^3+^ content change (between first and fifth cycle) and the Sm-doping ratio. This figure indicates that the addition of Sm (even at 5%) results in a significant decrease in the change of Ce^3+^ between the fresh and spent catalysts. The Ce^3+^ content for pure ceria decreases from 24.2% to 15.8%, suggesting poor stability performance. In contrast, changes in the Ce^3+^ content of Sm-doped ceria catalysts are below 1%, with 20% Sm doping only 0.5%. This indicates that Sm doping is beneficial for improving catalyst stability and maintaining activity. Figure 10 also shows that the relationship between the change of the O_α_ percentage (between first and fifth cycle) and the Sm-doping ratio. It can be observed that with an increase in the doping ratio, the decrease of the O_α_ percentage becomes smaller. The 5% and 10% Sm-doped catalysts decrease by about 2%, whereas the 20% Sm catalyst does not show any change. This suggests that the higher Sm doping makes a more stable catalyst and prevents the decline of the O_α_ percentage. The O_α_ percentage of the fresh CeO_2_ catalyst is 39.6% and yields to 29.5% after five cycles. This indicates that the OSC of the CeO_2_ catalysts is not stable, and the significant decline of OSC after reacting with soot leads to poor stability of pure ceria catalysts.

Moreover, the XRD results have shown that Sm is incorporated into the CeO_2_ lattice and causes lattice expansion. The increased crystallite size of the spent catalyst compared with the fresh one indicates that recrystallization has occurred. However, the larger crystallite size does not benefit the catalyst activity towards soot oxidation, as smaller crystallite size could favor oxygen diffusion and provide more contact points. The Sm-doped catalyst shows a smaller change in crystallite size after reactions, and such a difference becomes lesser with higher amounts of Sm doping, indicating that adding Sm can inhibit this recrystallization process. Therefore, adding Sm can stabilize the catalyst crystal structure and helps maintain surface properties, such as the Ce^3+^ and O_α_ percentages.

## 4. Conclusions

Doping 5%, 10%, and 20% samarium into a CeO_2_ lattice was achieved using the solution combustion synthesis (SCS) method to improve the stability of the nanostructured ceria-based catalyst for soot oxidation. In general, at a higher Sm content (10% and 20%), the reactivity (represented by T50) nearly plateaued after five cycles. However, increasing the Sm content was accompanied with a decrease in activity for soot oxidation. Considering the effects of Sm doping in both stability and activity, the 10% Sm-doped catalyst showed the best compromise between the catalyst stability and activity. Ce^3+^ and O_α_ were found to play an important role in controlling catalytic soot-oxidation activity, which agrees with the expectation that they are directly related to the oxygen vacancies and oxygen storage capacity of the catalyst. Sm-doped catalysts, especially at 10% and 20% Sm, showed a minimized decrease in Ce^3+^ content and O_α_ percentage between the fresh and spent catalysts after five cycles. Good stability in maintaining the crystallite size between the fresh and spent catalysts can also contribute to the greater stability for Sm-doped ceria catalysts. However, our experimental data did not indicate a clear correlation between the crystallite size and activity for these fresh and spent catalysts. Therefore, crystallite size growth is not considered here as a primary parameter for catalyst deactivation over cycling.

## Figures and Tables

**Table 1:** Texture results of Sm-doped ceria fresh and spent catalysts (after five cycles). Percentages for spent catalysts indicate relative changes compared to fresh catalysts.

Catalyst	Crystallite Size/nm	Lattice Parameter/Å	S_BET_/m^2^g^−1^
CeO_2_-fresh	12.33 (±0.09)	5.405 (±0.0018)	45.5 (±1.57)
Sm_0.05_Ce_0.95_-fresh	12.43 (±0.19)	5.424 (±0.0075)	42.2 (±0.99)
Sm_0.1_Ce_0.9_-fresh	11.89 (±0.17)	5.432 (±0.0028)	48.3 (±0.78)
Sm_0.2_Ce_0.8_-fresh	13.61 (±0.06)	5.442 (±0.0014)	40.9 (±1.94)
CeO_2_-spent	14.52 (±0.13)/+17.8%	5.418 (±0.0015)	40.9 (±1.43)/−10.1%
Sm_0.05_Ce_0.95_-spent	14.71 (±0.07)/+18.3%	5.430 (±0.0015)	40.5 (±0.75)/−4.0%
Sm_0.1_Ce_0.9_-spent	13.33 (±0.11)/+12.1%	5.436 (±0.0032)	46.2 (±2.36)/−4.3%
Sm_0.2_Ce_0.8_-spent	14.26 (±0.12)/+4.8%	5.445 (±0.0087)	40.2 (±1.52)/−1.7%

**Table 2:** Ce^3+^ percentages and O_α_ percentages of fresh and spent catalysts.

Catalyst	CeO_2_	Sm_0.05_Ce_0.95_	Sm_0.1_Ce_0.9_	Sm_0.2_Ce_0.8_
Ce^3+^ (%)-fresh	24.2	18.8	19.3	17.3
Ce^3+^ (%)-spent	15.8	17.9	18.5	16.8
O_α_ (%)-fresh	39.6	39.1	39.2	34.2
O_α_ (%)-spent	29.5	37.4	37.6	34.2

**Table 3:** I_554_/I_596_ of Raman results.

	Sm_0.05_Ce_0.95_	Sm_0.1_Ce_0.9_	Sm_0.2_Ce_0.8_
I_554_/I_596_ fresh	0.52	0.63	0.97
I_554_/I_596_ spent	0.69	0.73	1.06

## Supplementary Materials

The following are available online at https://www.mdpi.com/article/10.3390/nano12030392/s1: Figure S1. C1s spectra for all catalysts, Table S1. Peak position of Ce 3d spectra for fresh and spent catalysts.
