# Supplementary material for: Influence of Surface Properties of Nanostructured Ceria-Based Catalysts on Their Stability Performance
Source: Nanomaterials (Basel). 2022 Jan 25;12(3):392. doi: 10.3390/nano12030392 (PMC8839000; doi:10.3390/nano12030392)
Supplement: Supplementary file 1 [file nanomaterials-12-00392-s001.zip › nanomaterials-1528005-supplementary.pdf]

nanomaterials

MDPI

Supplementary Materials

Influence of Surface Properties of Nanostructured Ceria-Based Catalysts on Their Stability Performance

Boyu Li [1], Eric Croiset [1] and John Z. Wen [2,*]

[1] Department of Chemical Engineering, University of Waterloo, Waterloo, ON N2L3G1, Canada; b264li@uwaterloo.ca (B.L.); ecroiset@uwaterloo.ca (E.C.)
[2] Department of Mechanical & Mechatronics Engineering, University of Waterloo, Waterloo, ON N2L3G1, Canada
* Correspondence: john.wen@uwaterloo.ca; Tel: +1-519-888-4567 ext.38362

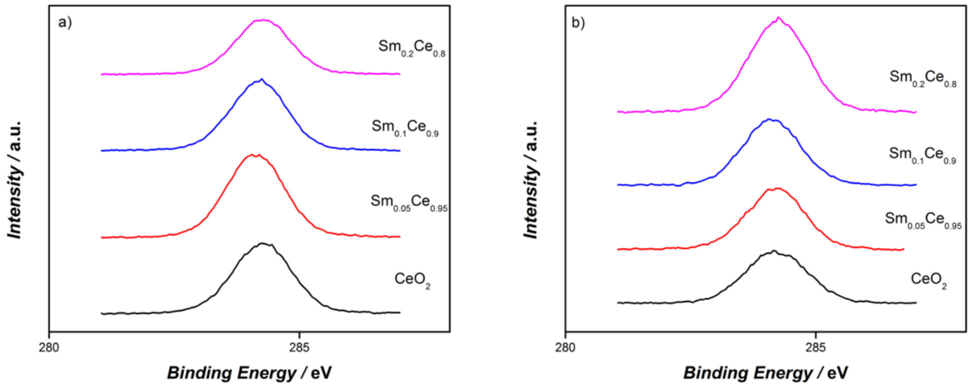

Figure S1. C1s spectra for all catalysts.

Table S1. Peak position of Ce 3d spectra for fresh and spent catalysts.

		v0 BE (eV)	v BE (eV)	v1 BE (eV)	v2 BE (eV)	v3 BE (eV)	u0 BE (eV)	u BE (eV)	u1 BE (eV)	u2 BE (eV)	u3 BE (eV)
Fresh Catalysts	CeO_2	879.8	882.4	885.1	888.7	898.6	898.3	900.9	903.4	907.5	917.0
	$Sm_{0.05}Ce_{0.95}$	879.9	882.3	885.0	888.8	898.5	898.2	900.8	903.3	907.5	916.8
	$Sm_{0.1}Ce_{0.9}$	880.1	882.2	884.6	888.5	898.6	898.2	900.7	903.0	907.4	916.8
	$Sm_{0.2}Ce_{0.8}$	880.2	882.2	884.3	888.5	898.7	898.3	900.6	902.4	907.1	916.7
Spent Catalysts	CeO_2	880.1	882.4	884.6	888.6	898.7	898.2	900.9	903.0	907.6	917.0
	$Sm_{0.05}Ce_{0.95}$	880.2	882.4	884.6	888.7	898.6	898.2	900.8	902.9	907.4	916.9
	$Sm_{0.1}Ce_{0.9}$	880.2	882.4	884.5	888.7	898.7	898.3	901.0	902.8	907.7	917.1
	$Sm_{0.2}Ce_{0.8}$	880.1	882.3	884.8	888.6	898.7	898.2	900.9	903.2	907.6	916.9
